# End digit preference in blood pressure measurement in a hypertension specialty clinic in southwest Nigeria

**DOI:** 10.5830/CVJA-2011-045

**Published:** 2012-03

**Authors:** OE Ayodele, OO Okunola, AA Akintunde, EO Sanya

**Affiliations:** Department of Medicine, Ladoke Akintola University of Technology, Osogbo, Osun State, Nigeria; Department of Medicine, Ladoke Akintola University of Technology, Osogbo, Osun State, Nigeria; Department of Medicine, Ladoke Akintola University of Technology, Osogbo, Osun State, Nigeria; Department of Medicine, University of Ilorin, Ilorin, Kwara State, Nigeria

**Keywords:** end digit preference, blood pressure measurement, hypertension specialty clinic, southwest Nigeria

## Abstract

**Background:**

One of the observer errors associated with blood pressure (BP) measurement using a mercury sphygmomanometer is end digit preference (EDP) which refers to the occurrence of a particular end digit more frequently than would be expected by chance alone. Published reports, mainly from outside Africa, have shown a high prevalence ranging from 22 to 90% of end digit zero in BP readings taken by healthcare workers (HCWs). This study examined the prevalence of EDP and patients’ and physicians’ characteristics influencing the occurrence of EDP.

**Methods:**

A retrospective review was undertaken of BP readings of 114 patients seen over a two-month period at our hypertension specialty clinic.

**Results:**

Nurses and physicians displayed a high frequency of preference for end digit zero in systolic blood pressure (SBP) and diastolic blood pressure (DBP) readings. The preference for end digit zero was, however, higher for nurses than for physicians (SBP: 98.5 vs 51.2%, *p* < 0.001; DBP: 98.5 vs 64.3%, *p* < 0.001). Among the physicians, the consultant staff displayed the least preference for end digit zero compared to resident doctors. There was no statistically significant difference in gender, age, weight, height and BMI of those with BP readings with end digit zero compared with those with non-zero end digits.

**Conclusion:**

The high prevalence of EDP for zero argues for the training, retraining and certification of HCWs in BP measurement and the institution of a regular monitoring and feedback system on EDP in order to minimise this observer error.

## Abstract

The toxicity of mercury notwithstanding, the mercury sphygmomanometer remains the most widely used apparatus for blood pressure (BP) measurement in Nigeria.^1^ Although protocols for measuring BP are well described and standardised,^2^-^5^ inaccuracies of measurement do occur from the use of faulty or malfunctioning equipment, improper technique, or observer errors or bias.^5^-^9^ Observer biases described in clinical practice and trials include duplication of previous BP recordings, rounding off to or below pre-set cut-off values for the diagnosis or control of hypertension and rounding off BP values to a particular end digit.^5^-^20^

End digit preference (EDP) refers to the occurrence of a particular end digit more frequently than would be expected by chance alone and it is a widely accepted indicator of low-quality BP measurement.^5^-^8^ In view of the fact that mercury sphygmomanometers are calibrated in increments of 2 mmHg, individual readings should only end in 0, 2, 4, 6 and 8.

If BP measurements are done strictly according to guidelines, the expected prevalence of each of the digits should be 20%. However, various studies have shown that there is an increased prevalence of end digit 0 (zero), ranging from 22 to 90%, depending on the clinical setting (primary healthcare, specialty clinic, drug-trial setting), the skill or qualification of the healthcare worker who took the BP, and the presence of feedback-monitoring systems for EDP.^10^-^20^

End digit preference leads to over- or underestimation of actual BP. Underestimation of BP could mean missing the diagnosis of hypertension in a patient, which can result in significant morbidity and mortality due to lack of treatment.^2^-^5^ On the other hand, overestimation of BP could result in inappropriate diagnosis of hypertension, inappropriate labelling, lifetime subjection to antihypertensive treatment with its attendant side effects, and reduction in quality of life and financial status due to loss of work or hospitalisation.^2^-^5^,^21^-^23^

Furthermore, EDP had been associated with difficulty in assessing associations between blood pressure and other potential cardiovascular risk factors by reducing the power of statistical tests.^10^ Therefore the diagnosis of hypertension, the eligibility for treatment, the assessment of adequacy of BP control, the recruitment for clinical trials on blood pressure and other cardiovascular risk factors and, by implication, the validity of the findings of such trials all depend on proper measurement and recording of BP.

There is a dearth of publications on EDP in BP measurement from Africa, despite the fact that one of the earliest publications on this clinical entity was from South Africa.^10^ We therefore conducted this study to determine the frequency of EDP in systolic (SBP) and diastolic blood pressure (DBP) readings taken by nurses and attending physicians in our hypertension speciality clinic. We also determined patients’ and physicians’ characteristics influencing the occurrence of EDP.

## Methods

We retrospectively reviewed the medical records of patients with hypertension attending the Hypertension Clinic of Ladoke Akintola University of Technology Teaching Hospital, Osogbo, Osun State, Nigeria over a two-month period (1 February to 31 March 2010). In our hypertension clinic, a patient’s blood pressure is first taken by a nurse using a mercury sphygmomanometer and the value is recorded in the patient’s folder. The physician repeats the BP measurement during the same visit as part of the clinical evaluation of the patient.

Only folders of patients with at least three clinic visits before the commencement of the study and with proper documentation of the BP values by nurses and doctors were selected for the study. The latest three BP readings by the nurses and doctors were extracted from the medical records of the patients.

The designation of the attending doctor at each clinic visit was also recorded. The nurses involved in BP measurements of patients in our clinic during the study period were registered nurses and they were in the same cadre. Ten doctors and two nurses were responsible for taking the readings included in this analysis.

Patients’ gender, age, height (m) and latest weight (kg) were recorded. Body mass index (BMI) was calculated from weight/height_2_ (kg/m_2_).

Ethical approval for the study was obtained from the Research Ethics Committee of Ladoke Akintola University of Technology Teaching Hospital, Osogbo, Osun State, Nigeria.

## Statistical analysis

Continuous variables were summarised as means ± standard deviation and categorical variables were displayed as percentages. Distribution of end digits of systolic and diastolic blood pressure values was noted and Chi-square comparison was performed to examine the significant differences in the occurrence of 0, 2, 4, 5, 6 and 8 end digits reported for both systolic and diastolic BP readings by nurses and doctors. Differences in zero and non-zero end digits of BP values by different cadres of doctors were also evaluated.

The last clinic attendance BP value was used to assess the demographic characteristics of patients that may influence EDP by physicians. The demographic parameters were compared using Chi-square for categorical and Student’s *t*-test for continuous variables in the group of patients with zero EDP and the group with non-zero end digits. All *p*-values less than 0.05 were considered to be statistically significant.

Statistical analysis was done using Statistical Package for Social Sciences (SPSS) software, version 15 (SPSS, Chicago, IL, USA). Figures were drawn using the Microsoft Office Excel 2007 version.

## Results

The study population consisted of 114 patients (37 males and 77 females) and their demographic features are shown in Table 1. The female preponderance of the study population (female:male was 2.1:1) reflects the clinic attendance of our patients throughout the year. The male patients were significantly older and taller than the females. However, the female patients had higher BMI than the males.

**Table 1:** Demographic And Clinical Characteristics Of The Study Population

*Patients’ characteristics*	*Male (n = 37)*	*Female (n = 77)*	*Total (n = 114)*	*Range*	p-*value*
Age (years)	68.1 ± 11.8	59.9 ± 11.9	62.5 ± 12.5	23–85	0.001
Weight (kg)	69.1 ± 11.3	71.5 ± 17.2	70.8 ± 15.5	43–120	0.438
Height (m)	1.69 ± 0.06	1.60 ± 0.06	1.63 ± 0.07	1.46–1.81	< 0.001
BMI (kg/m^2^)	24.4 ± 4.6	28.1 ± 6.2	26.9 ± 6.0	15.8–42.8	0.001
Nurses’ mean SBP (mmHg)	132.7 ± 23.9	136.0 ± 22.5	134.9 ± 22.9	90–210	0.479
Doctors’ mean SBP (mmHg)	136.9 ± 24.6	140.4 ± 25.1	139.3 ± 24.9	90–230	0.488
Nurses’ mean DBP (mmHg)	78.9 ± 16.6	82.8 ± 14.0	81.5 ± 15.0	50–130	0.195
Doctors’ mean DBP (mmHg)	80.0 ± 12.3	80.5 ± 13.5	80.3 ± 13.0	54–120	0.835
Mean nurse–doctor SBP difference	–4.22 ± 20.19	-4.42 ± 20.49	-4.36 ± 20.31	–60 to +32	0.959
Mean nurse–doctor DBP difference	–1.03 ± 12.63	2.31 ± 13.46	1.22 ± 13.23	–44 to 40	0.209

There was no statistically significant difference in the mean SBP and DBP of males and females. The means of the nurse–doctor SBP difference and nurse–doctor DBP difference were –4.36 mmHg (range –60 to +32 mmHg) and 1.22 mmHg (range–44 to +40 mmHg), respectively.

The nurses and the doctors took 342 BP readings each. Table 2 shows the distribution of the end digits of the systolic and diastolic blood pressure readings. The distribution of end digits for SBP readings by nurses revealed that 98.5% ended in the digit 0; 0.3% ended in the digit 4; and 1.2% in the digit 5. On the other hand, 51.2% of the SBP readings of doctors ended in the digit 0; 14.3% in the digit 4; and 16.4% in the digit 6. None of the doctors’ readings ended with the digit 5.

**Table 2:** Distribution Of End Digits Of Systolic And Diastolic Blood Pressure Readings By Doctors And Nurses

	*Systolic blood pressure readings*		*Diastolic blood pressure readings*	
*End digit*	*Nurses (%)*	*Doctors (%)*	*Nurses (%)*	*Doctors (%)*
0	337 (98.5)*	175 (51.2)	337 (98.5)**	220 (64.3)
2	0 (0)	33 (9.6)	0 (0)	15 (4.4)
4	1 (0.3)	49 (14.3)	1 (0.3)	46 (13.5)
5	4 (1.2)	0 (0)	1 (0.3)	0 (0)
6	0 (0)	56 (16.4)	0 (0)	32 (9.4)
8	0 (0)	29 (8.5)	3 (0.9)	29 (8.5)
Total	342 (100)	342 (100)	342 (100)	342 (100)

*χ^2^ = 219.3, *p* < 0.001; ** χ^2^ = 136.7, *p* < 0.001.

Nurses had a statistically significantly higher occurrence of SBP zero EDP compared to doctors (*p* < 0.001). For DBP, 98.5% of nurses’ readings ended in the digit 0 while 64.3% of doctors’ readings ended in the digit 0 (*p* < 0.001).

**Fig. 1:**
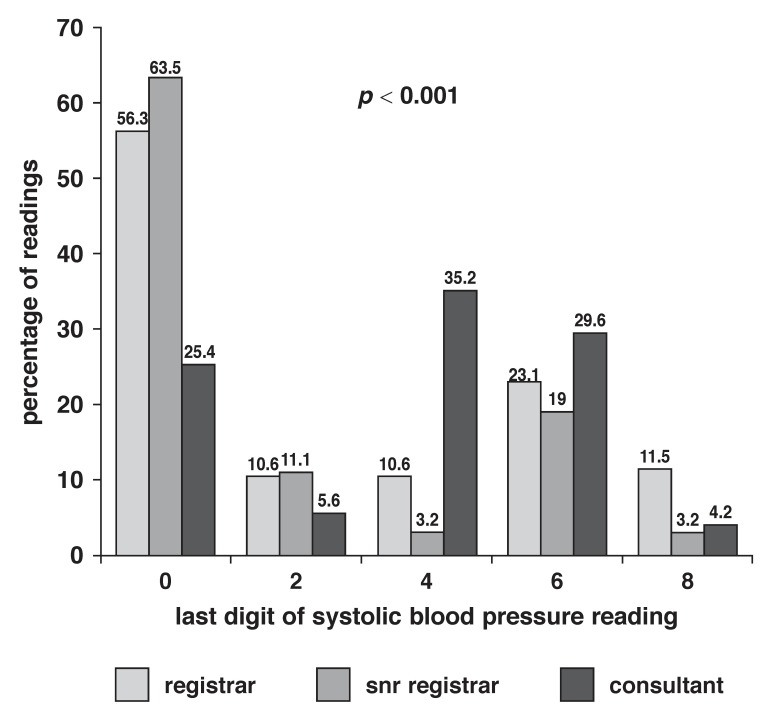
Percentage of systolic blood pressure end digit values among different cadres of doctors.

**Fig. 2:**
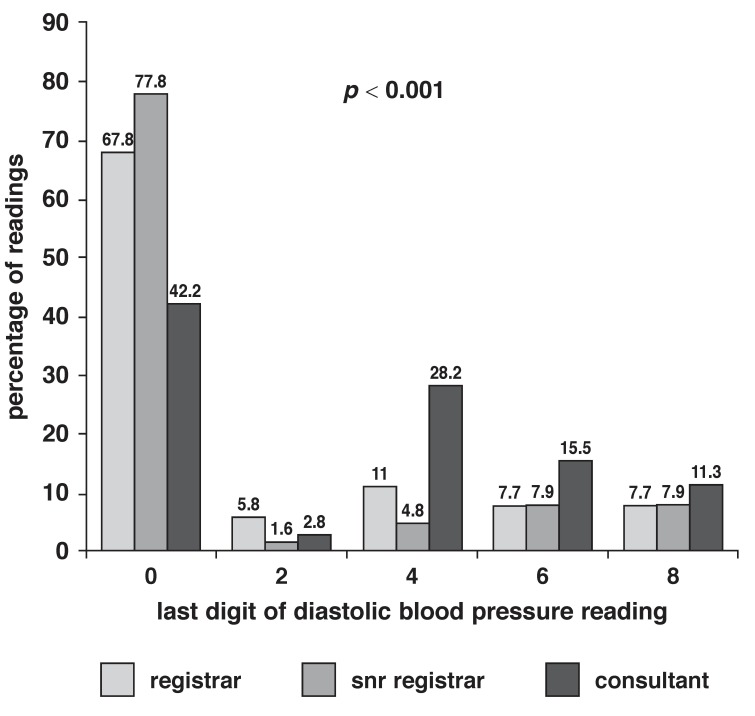
Percentage of diastolic blood pressure end digit values among different cadres of doctors.

Figs 1 and 2 show the distribution of EDP for SBP and DBP readings, respectively, among different cadres of doctors. The consultant staff showed the least preference for end-digit 0 when compared with registrars and senior registrars. While 25.4% of the SBP readings by consultant staff ended in the digit 0, 56.3% of the readings by registrars and 63.5% of those by senior registrars ended in the digit 0 (*p* < 0.001). Although the consultant staff recorded a higher frequency of end digit 0 for DBP than SBP (42.3%), the frequency of end digit 0 was still lower than that by registrars (67.8%) and senior registrars (77.8%), and this difference was statistically significant (*p* < 0.001).

A comparison of the characteristics of the patients was made in those whose SBP and DBP readings ended with end digit 0 and those with non-zero end digits (Table 3). There was no significant difference in the gender, and mean age, weight, height and body mass index of those with end digit 0 compared with those with non-zero end digits.

**Table 3:** Characteristics Of Patients By End Digits Of Blood Pressure Readings

	*SBP readings end digits*			*DBP readings end digits*		
*Patients’ characteristics*	*Zero (%)*	*Non-zero (%)*	p-*value*	*Zero (%)*	*Non-zero (%)*	p-*value*
Gender						
Male	17 (45.9)	20 (54.1)	0.465	24 (64.9)	13 (35.1)	0.519
Female	41 (53.2)	36 (46.8)		46 (59.7)	31 (40.3)	
Mean age (years)	61.9 ± 11.7	63.2 ± 13.2	0.569	62.9 ± 11.4	62.0 ± 14.1	0.718
Mean weight (kg)	68.99 ± 16.66	72.63 ± 14.18	0.214	71.4 ± 16.8	69.8 ± 13.3	0.599
Mean height (m)	1.63 ± 0.07	1.63 ± 0.07	0.878	1.63 ± 0.07	1.63 ± 0.08	0.897
Mean BMI (kg/m^2^)	26.2 ± 5.9	27.6 ± 6.0	0.253	26.9 ± 6.5	26.8 ± 5.0	0.908
Mean BP (mmHg)	139.5 ± 29.9	139.0 ± 18.5	0.924	80.4 ± 13.6	80.1 ± 12.3	0.908

BMI – body mass index, BP – blood pressure.

## Discussion

A zero EDP of 98.5% of SBP and DBP readings by nurses in our clinic was much higher than the 22.2–40.8% of SBP and 21.8–53.6% of DBP readings by hypertension nurse specialists at the Mayo Clinic, Rochester, as reported by Graves *et al.*^17^ These differences in the frequency of zero EDP may be due to the differences in the degree of training of our nurses and those of the Mayo Clinic, since our nurses are not specially trained to run the hypertension clinic. Our findings argue for specialisation or retraining of the nurses involved in running the hypertension clinic in order to improve their clinical competence in BP measurement and documentation.

The zero EDP of 51.2% of SBP and 64.3% of DBP readings by doctors in our clinic was much higher than the expected 20%. Although a zero EDP of 51.2% of SBP readings was lower than the 60–84% reported in most clinical practice-based studies,^8^,^14^-^16^ we expected a lower frequency than this. This assumption was based on the premise that doctors working in the hypertension specialty clinic should have acquired clinical competence in BP measurement.

The zero EDP of 64.3% of DBP readings in our study is similar to the 64% obtained by Kim *et al.*,^14^ the 65% by Patterson *et al.*,^8^ and the 62% by Broad *et al.*,^15^ but higher than the 36% obtained by Thavarajah *et al.*^11^ in a similar hypertension specialty clinic setting.

Subgroup analysis of the readings by doctors showed that the consultant staff had the least preference for end digit zero in their BP measurements, although the findings of a zero EDP of 25.4% of SBP and 42.3% of DBP were higher than the expected 20%. The lower frequency of zero EDP in the BP readings by consultants was not unexpected, taking into consideration their years of training and practice, and the fact they are more likely to be conversant with guidelines for BP measurement.

The senior residents showed a higher frequency for zero EDP than the junior residents, a finding which was unexpected, considering the senior residents’ knowledge base and years of training. However, many publications have pointed out the lack of formal training and assessment in BP measurement during undergraduate and postgraduate medical training, and this could have been responsible for our findings.^9^,^19^,^24^-^26^ In fact, Gonzalez-Lopez *et al.*^24^ pointed out that inadequate knowledge of BP measurement is unlikely to improve during specialised postgraduate training since the skill is not taught during this period because of the assumption that the relevant skill should have been acquired earlier on during undergraduate training.

We did not find any impact of patient-related factors such as gender, age, weight and BMI on the occurrence of EDP. Our results differ from earlier reports by Graves *et al.*,^17^ who found an association between increasing age and zero EDP for DBP, and the findings by Kim *et al.*,^14^ who documented greater EDP for SBP in older patients and women in BP taken by non-physicians, and greater EDP for DBP in less-obese patients in BP taken by physicians.

Publications abound in the literature on the debate over whether mercury sphygmomanometers should be phased out in clinical practice.^27^,^28^ However, it is likely that mercury manometers will remain in use in clinical settings in Nigeria and many parts of Africa for a long period of time. This is because of their low cost of purchase and maintenance (no need for electricity or battery), their simple design (a simple gravity-based unit with easy calibration), their arguably infrequent need for repair, their validation in many clinical circumstances against direct intra-arterial BP measurement, and the rarity of reported health problems associated with exposure to elemental mercury enclosed in sphygmomanometers.^1^,^28^,^29^

Although the use of automated BP measuring devices may help in eliminating EDP,^12^,^29^ the recommendation by some of the manufacturers of these devices to recalibrate them against a mercury manometer every six months, and the non-elimination by these devices of other sources of errors in BP measurement, such as choice of cuff size, placement of cuff, posture of subject, arm support, etc, argue against the total abandonment of mercury sphygmomanometers in clinical settings.^28^

Studies have also shown that training, retraining and certification of healthcare workers in BP measurement with regular monitoring and feedback on end digit and number preference help to reduce or eliminate end digit and other observer bias and errors associated with BP measurement.^12^,^17^,^18^,^28^

Our cohort consisted of patients being managed for hypertension, and good BP control can be defined as SBP < 140 mmHg and/or DBP < 90 mmHg. Only two (3.8%) out of 53 patients with SBP < 140 mmHg had SBP of 138 mmHg and two (2.4%) out of 84 patients with DBP < 90 mmHg had DBP of 88 mmHg. Therefore, the occurrence of an 8 as end digit was not related to treatment thresholds.^2^-^4^

The means of the difference in the nurse–doctor SBP measurements was –4.36 mmHg, indicating that the SBP readings by the nurses were on the average lower than those by doctors. However, the nurse–doctor DBP difference of 1.22 indicates that DBP recorded by the nurses were on the average higher than that recorded by doctors.

The means and range of the nurse–doctor SBP differences in our study of –4.36 mmHg and –60 to +32 mmHg was comparable to –6.3 mmHg and –67 to +66 mmHg reported by La Batide-Alanore *et al.*^30^ from France. Although the mean nurse–doctor DBP difference in our study was higher than that reported by La Batide-Alanore *et al.* (1.22 vs –7.9 mmHg), the ranges of the difference were similar (–44 to +40 vs –44 to +31 mmHg).^30^

This study had a few limitations. The high frequency of zero EDP in our study is an indication that the various healthcare workers involved in this study were rounding the BP readings to the nearest 10 mmHg. In view of the retrospective nature of the study, we could not tell whether the readings were rounded down or up. Second, we had few missing data on weight (one subject) and height (nine subjects). Third, in view of its retrospective nature, we could not assess whether the BP measurements were done according to the guidelines on BP measurement. Lastly, although the number of our observations may appear small, most publications on this subject with larger number of observations involved multiple centres with electronic storage of BP readings.^12^,^14^-^16^,^18^,^20^,^29^

## Conclusion

We found a high frequency of zero EDP in BP measurements in our hypertension specialty clinic, particularly from among the nursing staff and resident doctors. Our findings argue for the training, retraining and certification of nurses and doctors in BP measurement and the institution of a regular monitoring and feedback system on EDP in order to minimise this observer error.
